# Visual Contrast Modulates Operant Learning Responses in Larval Zebrafish

**DOI:** 10.3389/fnbeh.2019.00004

**Published:** 2019-01-24

**Authors:** Wenbin Yang, Yutong Meng, Danyang Li, Quan Wen

**Affiliations:** ^1^Hefei National Laboratory for Physical Sciences at the Microscale, Center for Integrative Imaging, School of Life Sciences, University of Science and Technology of China, Hefei, China; ^2^Chinese Academy of Sciences Key Laboratory of Brain Function and Disease, Hefei, China; ^3^Center for Excellence in Brain Science and Intelligence Technology, Chinese Academy of Sciences, Shanghai, China

**Keywords:** zebrafish larvae, behavioral neuroscience, learning, vision, high-throughput imaging, automated image analysis

## Abstract

The larval zebrafish is a promising vertebrate model organism to study neural mechanisms underlying learning and memory due to its small brain and rich behavioral repertoire. Here, we report on a high-throughput operant conditioning system for zebrafish larvae, which can simultaneously train 12 fish to associate a visual conditioned pattern with electroshocks. We find that the learning responses can be enhanced by the visual contrast, not the spatial features of the conditioned patterns, highlighted by several behavioral metrics. By further characterizing the learning curves as well as memory extinction, we demonstrate that the percentage of learners and the memory length increase as the conditioned pattern becomes darker. Finally, little difference in operant learning responses was found between AB wild-type fish and *elavl3:H2B-GCaMP6f* transgenic fish.

## Introduction

In operant conditioning, an animal learns to correlate its behavioral responses with consequences. Responses leading to satisfying consequences are reinforced whereas those leading to negative consequences are weakened or discarded. This form of associative learning has been intensively studied on behavioral, cellular, and molecular levels (Freund and Walker, 1972; Brembs et al., 2002; Nargeot and Simmers, 2011; Ishikawa et al., 2014), and many factors, such as dopaminergic signaling (Wise, 2004; Wassum et al., 2011; Steinberg et al., 2013) and Hebbian plasticity (Bi and Poo, 1998; Cassenaer and Laurent, 2007; Froemke et al., 2010), are known to play critical roles. Nevertheless, it remains largely elusive how *in vivo* learning rules, by which local synaptic plasticity and reward signaling must be integrated across distributed brain circuits, subserve adaptive animal behaviors. To make progress, it would be illuminating to measure neural activity of defined cell types at the whole-brain scale during the entire learning process.

The larval zebrafish is a promising vertebrate model to identify brain-wide mechanisms underlying learning and memory: its small brain is a great compromise between system complexity and simplicity. Recently, it has become possible to perform whole-brain imaging of calcium activity in freely behaving larval zebrafish (Cong et al., 2017; Kim et al., 2017). Whereas, fish are well-established animal models to study learning and memory (Davis and Agranoff, 1966; Agranoff and Davis, 1968), few associative learning paradigms have been developed for zebrafish larvae. Li (2012) reported operant learning in head-fixed larvae, in which fish learned to correlate the relief of aversive heat stimulus with biased tail turning. Valente et al. (2012) showed that 1-week larvae were unable to perform an operant learning paradigm, in which fish must learn to swim to the other half of an arena to avoid electroshocks. Other reports demonstrated that larval zebrafish could be classically conditioned: they could associate the conditioned stimulus (CS)—a moving spot with the unconditioned stimulus (US)—a touch of the body (Aizenberg and Schuman, 2011). Social reward, such as visual access to conspecifics, could also be paired with a distinct visual environment cue during classical conditioning in larval zebrafish (Hinz et al., 2013).

Zebrafish have sophisticated vision. Adult zebrafish can distinguish colors (Colwill et al., 2005; Zimmermann et al., 2018) and visual patterns with different orientations (Colwill et al., 2005). Spatial and non-spatial visual learning tasks have been studied in adult zebrafish (Arthur and Levin, 2001). The visual system of zebrafish develops rapidly. 70–80 hpf (hour after post-fertilization) larval zebrafish can respond to abrupt light intensity change (Easter and Nicola, 1996), and exhibit optokinetic responses to rotating illuminated stripes (Huang and Neuhauss, 2008; Portugues and Engert, 2009; Mueller and Neuhauss, 2010). Seven-days-old larval zebrafish show strong optomotor responses to sophisticated motion stimuli (Orger et al., 2000; Roeser and Baier, 2003; Orger and Baier, 2005). However, much less is known about how properties of visual stimuli would modulate learning process in larval zebrafish.

Here, we report a modified operant conditioning paradigm (Valente et al., 2012) in freely swimming larval zebrafish, a system that combines a high-throughput automated training process and a toolkit for post-data analysis and storage. We use our new paradigm to investigate how visual contrast modulated the operant learning responses in larvae, characterized by both the positional and turning metrics. The measurements of learning curves provide a way to investigate memory extinction in larvae zebrafish. Moreover, we compare the differences between wild-type and transgenic fish in operant learning responses and memory extinction. We demonstrate that learning responses can be enhanced by a visual conditioned pattern that is darker than the background and that the percentage of learners as well as the memory length increase with darker conditioned patterns.

## Materials and Methods

### Ethical Statement of Animals-Using

Handling and care of all animals were conducted in strict accordance with the guidelines and regulations set forth by University of Science and Technology of China (USTC) Animal Resources Center, and University Animal Care and Use Committee. Both raising and training protocols were approved by the Committee on the Ethics of Animal Experiments of the USTC (permit number: USTCACUC1103013).

### Animals and Raising

Zebrafish (*Danio rerio*) of the genotype *elavl3:H2B-GCaMP6f* and AB wild-type fish were used in all experiments. All tested fish were 7–10 dpf (day past fertilization) larvae. They were bred, raised, and housed in the same environment. Fish were fed two times per day from 6 dpf with paramecium in the morning (8–9 a.m.) and evening (6–7 p.m.) before being used in experiments. Water was replaced with E2 medium (Cunliffe, 2003) in the morning (8–9 a.m.) and evening (6–7 p.m.). Water temperature was maintained at 28.5°C. Light was turned on at 08:00 a.m. and off at 10:00 p.m.

### Experimental Setup

**Figure 1:**
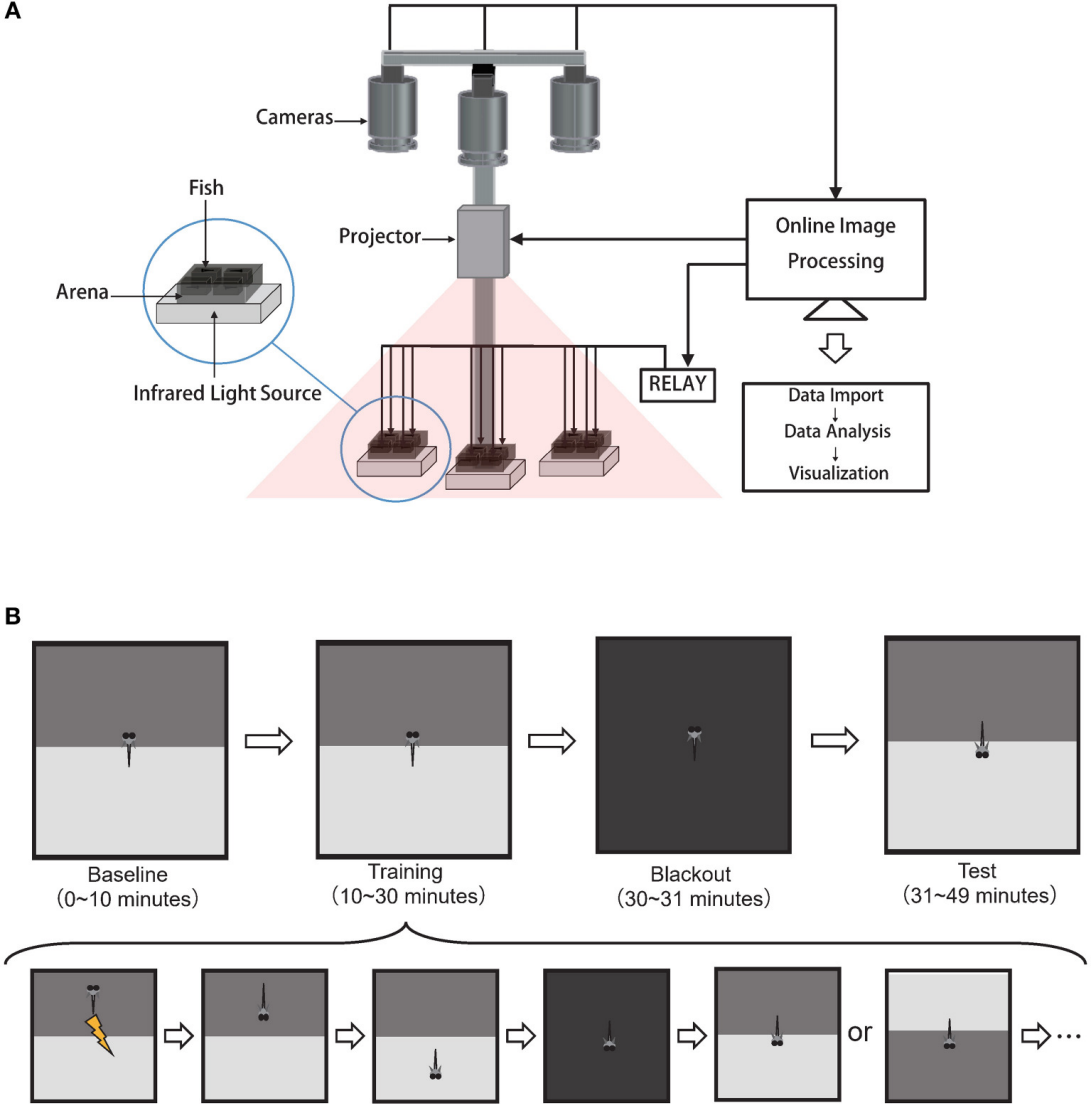
High-throughput automated behavioral training system for acquiring and analyzing operant conditioning responses in larval zebrafish. **(A)** Schematics of the behavioral system. Each arena held one fish. Cameras, projector, relay were controlled by the custom software BLITZ. Custom software ABLITZER imported the BLITZ-produced behavioral data, analyzed them, and visualized the results. **(B)** The operant conditioning paradigm: top is the experiment procedure and bottom is the detailed steps during the training phase.

The behavioral system with custom software suites and supporting hardware was built to achieve an end-to-end high-throughput experimental workflow (Figure 1A).

#### Hardware

Zebrafish swam freely in custom-built acrylic containers with transparent bottoms. Each container was divided into four arenas separated by opaque walls. The arena's size is 3 cm × 3 cm × 1 cm, with water filled (Supplementary Figure 7). Each arena held one fish. Three CMOS cameras (Basler aca2000-165umNIR, Germany) with adjustable lens (Canon, Model EF-S 18-55mm f/3.5-5.6 IS II, Japan) simultaneously captured swimming behaviors at 10 frames per second. Three infrared LEDs (Kemai Vision, China, model HF-FX90, wavelength 940 nm) illuminated each container from below. A 700 nm long-pass filter (Thorlabs FEL0700, US.) was positioned in front of each camera to block visible light and to facilitate online imaging processing with custom software BLITZ. Visual stimuli were projected onto three containers (PIQS Projector S1, 14.6 × 7.85 × 1.75 cm, 854 × 480 pixels). Electroshocks (100 ms, 9 Volt/3 cm) were delivered via two platinum filaments, one on each side of the arena. Shock delivery at each arena was controlled by custom software BLITZ via a 16-channel relay (HongFa JQC-3FF, China). Room temperature was controlled by an air-conditioner at 27°C.

#### Software Suites

Custom C++ software BLITZ (Behavioral Learning In The Zebrafish), which inherited the coding style from MindControl (Leifer et al., 2011), processed three video streams in parallel to obtain real-time head, center, tail positions and heading angle by using the Pylon library (Basler AG, Germany) and the open source computer vision library (OpenCV) (Bradski, 2000). The program also rendered visual pattern and programmable electroshocks delivery based on the timeline and real-time fish motion parameters. Experimental information (e.g., experiment start time, visual pattern index, electroshock delivery information, and fish motion parameters) were recorded in YAML files. Raw videos were recorded.

The BLITZ software is available at https://github.com/Wenlab/BLITZ.

Another custom MATLAB (The MathWorks, Inc.) software ABLITZER (the Analyzer of BLITZ Results) was used to import YAML files, to visualize data, as well as to perform the behavioral and statistical analysis.

The ABLITZER software is available at https://github.com/Wenlab/ABLITZER.

### Experimental Procedure

Fish were fed at least an hour before being used in the experiment. Fish were placed via a Pasteur pipette (Nest, US) from the raising tank to the experimental arenas. The behavioral experiment would not run until fish started moving around to avoid startle responses to novel stimuli. Fish in the paired-group were trained first with the self-control protocol (see below), and then with the operant conditioning protocol. Fish in the unpaired-group were trained first with the self-control protocol, and then with the unpaired operant conditioning protocol (see below).

Fish used in the paired-group and unpaired-group were all naive fish.

#### Operant Conditioning Protocol

This operant conditioning protocol was modified from Valente's learning paradigm (Valente et al., 2012). Here, fish would experience four different phases in order: baseline phase, training phase, blackout phase, and test phase (Figure 1B).

First, in the 10-min baseline phase, the visual pattern beneath each arena would flip between the CS at the top (Supplementary Figures 5A,C,E) and CS at the bottom (Supplementary Figures 5B,D,F) with a random duration that was uniformly sampled from 30 to 45 s.

Second, in the 20-min training phase, both the update of visual patterns and the delivery of electroshocks were dependent upon fish's behavior. After the visual pattern was updated (including the first visual pattern in the training stage), there was a 7-s delay for fish to make decisions before electroshocks were delivered based on their positions. If fish were in the CS zone after the delay time, whole-arena shocks would be delivered every 3 s until fish escaped from the CS zone. After fish stayed in the Non-CS zone for 48 s, the visual pattern (CS zone at the top or bottom) would update with equal probability. The whole procedure would repeat (Figure 1B bottom).

After the training phase, there was a 1-min blackout phase to deprive all visual stimuli.

Finally, in the last 18-min test phase, to ask whether fish could develop the association between the CS and US, the visual pattern interchanged every 2 min between the CS at the top and CS at the bottom.

#### Self-Control Conditioning Protocol

All phases were identical to the operant conditioning protocol, except for no electroshock delivery.

#### Unpaired Operant Conditioning Protocol

All phases were identical to the operant conditioning protocol except for the training phase, in which electroshocks, without pairing with visual patterns, were randomly delivered across the 20-min duration.

### Behavioral Analysis

#### Visual Contrast

The visual contrast was defined as the grayscale value difference between the conditioned pattern and the background pattern (pure-gray) (see Table 1 for more details). The light irradiance was measured with a power meter (PM16-130, Thorlabs) that averaged the received light over 50 s.

**Table 1:** Visual contrasts of all conditioned patterns.

	**Mean RGB value**	**Grayscale value**	**Visual contrast**	**Light irradiance (μW/cm^**2**^)**
Grayscale 0	(0, 0, 0)	0	−128	9.9
Grayscale 32	(32, 32, 32)	32	−96	28.9
Grayscale 43	(43, 43, 43)	43	−85	30.8
Grayscale 64	(64, 64, 64)	64	−64	36.9
Grayscale 96	(96, 96, 96)	96	−32	53.7
Red-black checkerboard	(128, 0, 0)	43	−85	40.7
White-black checkerboard	(128, 128, 128)	128	0	102.4
Background (grayscale 128)	(128, 128, 128)	128	0	108.1

*In the table, the first column lists all conditioned patterns used in the experiments. Visual contrast = grayscale value of conditioned pattern—grayscale value of background (128)*.

#### Pre-screening

We defined data quality as the percentage of non-frozen frames. Frames were considered frozen when fish did not move for over 1 s. Fish with data quality lower than 0.95 were excluded from the analysis since those fish did not swim spontaneously and frequently. Fish with poor data quality were considered unhealthy.

The positional index was defined as the percentage of frames when fish were in the non-CS zone.

#### Turning Analysis

We scored a turning event when the heading-angle-change between two consecutive frames exceeded 15 degrees. Fish would get +1 score when performing an escape turn, and −1 score when returning to the CS zone. Fish in the Non-CS zone executed an escape turn when they approached the midline (within twice body length) and then turned back (Supplementary Figure 6). The turning index was defined as

$$\begin{array}{l} turning\;index=\frac{1}{2}+\frac{s\left(+\right)+s\left(-\right)}{\left(\left|s\left(+\right)|+|s\left(-\right)\right|\right)\cdot 2} \end{array}$$

where, s(+) and s(–) are positive and negative scores, respectively. In this way, the turning index would fall between 0 and 1, the same range as the positional index.

#### Distance to the Mid-line

This was defined as a signed Euclidean distance from the fish head position to the mid-line. The sign was −1 when fish were in the CS zone and +1 when fish were in the non-CS zone.

#### Learning Analysis

To evaluate whether fish learned the operant conditioning task, we divided the entire operant conditioning protocol time into 24 2-min-epochs. The learning responses diminished in the absence of electroshocks during the test phase, which is known as memory extinction (Myers and Davis, 2007). The extinction point was computed as the first time when the positional index within an epoch dropped below the baseline. The recall period was defined from the starting time of test phase to the extinction point. Here we use memory length or recall period interchangeably. If the positional indices in the recall period were significantly higher than those in the baseline phase, fish were classified as learners (The unpaired *t*-test was applied). The extinction rate was defined as

$$\begin{array}{l} extinction\;rate=\frac{PI_{1}-PI_{2}}{Memory\;Length} \end{array}$$

where, PI_1_ is the positional index of all learners of the first peak at the beginning of test phase, PI_2_ is the positional index of all learners of the nearest valley to the extinction point.

The positional index increase is the difference between the mean positional index in the recall period and the mean index in the baseline period; the turning index increase is the difference between the mean turning index in the recall period and the mean index in the baseline period.

### Statistical Analysis

The paired *t*-tests were used to compare the difference of the same fish between different phases in the same conditioning protocol; whereas the unpaired *t*-tests were used for the comparison between fish trained with the unpaired operant conditioning protocol and those with operant conditioning protocol. The sample size exceeded 20 for all tests.

## Results

### Larval Zebrafish Show Significant Learning Responses in an Operant Conditioning Task

In our modified operant conditioning task (Figure 1B), larval zebrafish Tg (*elavl3:H2B-gcamp6f*) freely swam in an arena divided by two distinct patterns, each of which was projected onto one half of a transparent floor. In all cases, a pure-gray (grayscale-128) visual pattern was presented on the non-CS zone, and the CS patterns were presented on the other half. The CS was paired with the US—moderate electroshocks. The delivery of US and the update of visual patterns depended upon fish's positions and time (see Materials and Methods for detailed experimental procedures).

To scale up the training process, we developed a high-throughput operant conditioning system (Figure 1A) with custom supporting software suites BLITZ and ABLITZER (see Materials and Methods) that allowed training twelve fish simultaneously. BLITZ provided a fully automated workflow from video capture, online image processing, to visual stimuli presentation and electroshocks delivery for all behavioral protocols. Raw experimental data were then imported, analyzed and visualized by ABLITZER.

**Figure 2:**
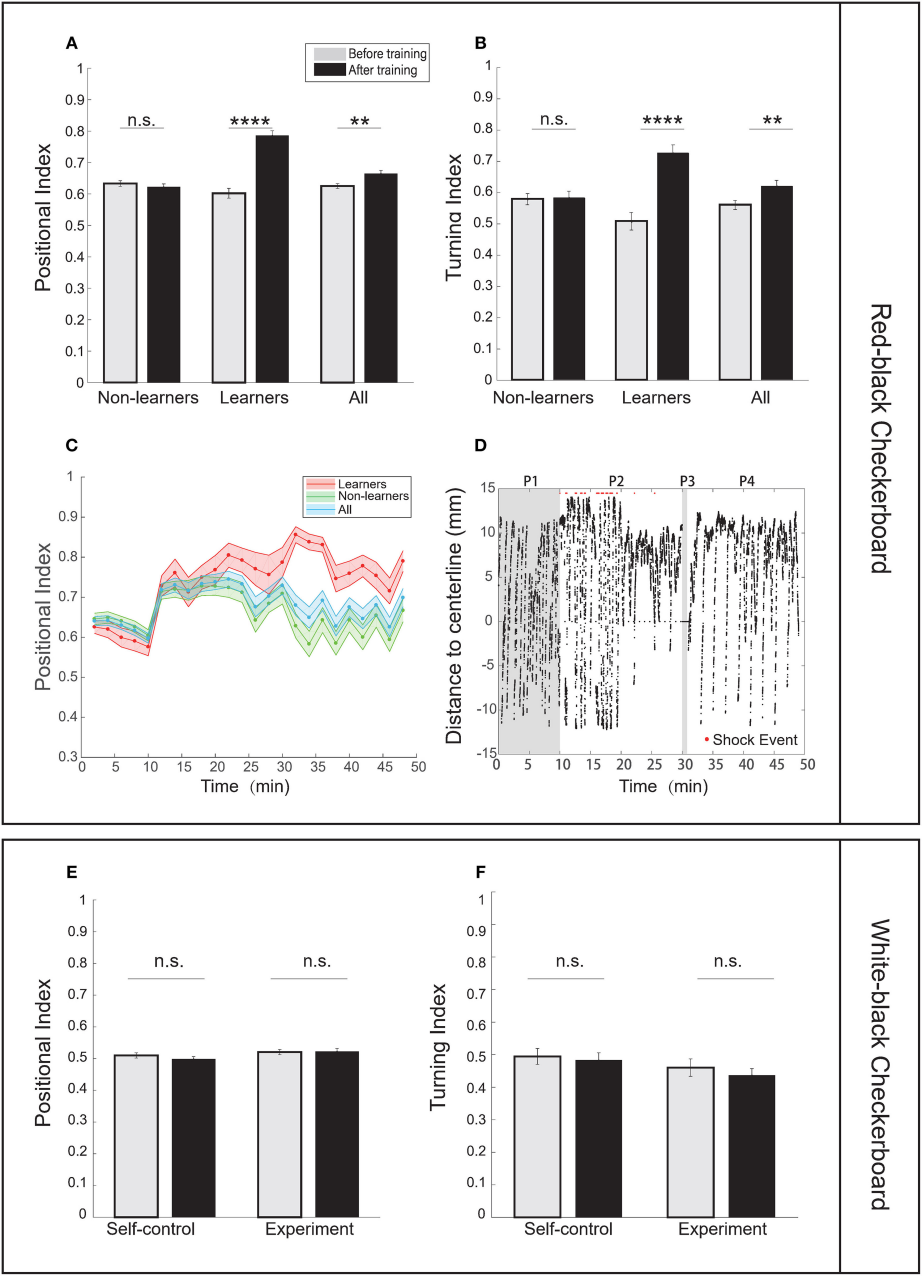
Zebrafish larvae showed enhanced learning responses in an operant conditioning task. **(A)** Analysis of the positional index suggests that learners showed significant enhancement of learning responses after training, whereas non-learners did not (CS zone was the red-black checkerboard pattern; *t*-test, *p* = 6.52e-12 for learners, *p* = 0.3849 for non-learners, and *p* = 0.0020 for all fish). **(B)** Learners also showed significant differences in the turning index (*t*-test, *p* = 2.28e-6 for learners, *p* = 0.8606 for non-learners, and *p* = 0.0099 for all fish). **(C)** Learning curves using the positional index in the experiment group. Fish were classified as learners (red shaded line) and non-learners (green shaded line). The entire training process was divided into 2-min epochs (CS zone: red-black checkerboard). **(D)** A typical learner's behavioral trace, characterized by the relative position to the midline. The red-black checkerboard conditioned pattern was presented to the animal. A positive distance implies fish staying in the non-CS zone (also see section Materials and Methods). Each red dot represents the delivery of one electroshock. (P1: baseline phase; P2: training phase; P3: blackout phase; P4: test phase). **(E)** Analysis of the positional index suggests that fish did not show significant learning responses when the white-black checkerboard was presented as the conditioned pattern (*t*-test, *p* = 0.8832 for the experiment group, *p* = 0.2493 for the self-control group). We found that only one fish could be classified as learner. Because no significant learning response was found in the experiment group, we did not carry out unpaired-control experiments. **(F)** Analysis of the turning index suggests that fish did not show significant learning responses (*t*-test, *p* = 0.3750 for the experiment group, *p* = 0.7089 for the self-control group. There was no unpaired-control group because no significant learning response was found in the experiment group). All error bars are SEM. **p < 0.01, ****p < 0.0001.

First, we tested several stimulus patterns in our operant conditioning task. When the red-black checkerboard was used as the conditioned pattern, 7–10 dpf zebrafish larvae showed significant learning responses (Figures 2A,B, Supplementary Videos 1, 2), evaluated based on two metrics—the positional index and turning index (see Material and Methods). By analyzing individual fish behavior, we classified 27 out of 104 (26%) fish as learners (Table 2) (see section Materials and Methods for criteria). Learners showed significant increase in the poisitional and turning indices after training, whereas non-learners did not (Figures 2A,B).

**Table 2:** Percentages of learners in AB wild-type and transgenic fish.

	**Grayscale 0**	**Grayscale 32**	**Grayscale 43**	**Grayscale 64**	**Grayscale 96**	**Red-black checkerboard**	**White-black checkerboard**
*elavl3*	21/44 (50%)	11/39 (28%)	9/39 (23%)	8/38 (21%)	1/33 (3%)	27/104 (26%)	1/37 (3%)
*AB/WT*	22/44 (50%)	8/36 (22%)	10/46 (22%)	11/44 (25%)	1/41 (2%)	17/68 (25%)	1/16 (6%)

*The numerator is the number of learners and the denominator is the total number of fish*.

Larval zebrafish have an innate positive light preference. Therefore, we developed two control settings: the self-control conditioning protocol in which no electroshock was delivered and the unpaired operant conditioning protocol in which electroshocks were randomly delivered (see Materials and Methods). Results from the two control settings were compared with those from the operant conditioning protocol to determine whether fish established the association (Supplementary Figures 1I,J). Figure 2C shows how the learning curves of learners, non-learners, and all fish, characterized by the positional index, changed during the entire learning process. Figure 2D shows a typical trajectory of a learner who tended to avoid the conditioned visual pattern after training.

We asked whether spatial features of the checkerboard alone could induce learning responses. However, when using the white-black checkerboard as the conditioned pattern, we found that fish showed little learning response after training (Figures 2E,F).

### Visual Contrast Modulates the Percentage of Learners in the Operant Conditioning Task

We asked whether visual contrast—the grayscale value difference between a conditioned pattern and background (grayscale-128)—would modulate learning. Because light irradiance from the projector increases with the grayscale value of an image (Table 1 and Supplementary Figure 4), we tested whether conditioned patterns with varying grayscale values (0, 32, 43, 64, and 96) would modulate operant learning responses.

**Figure 3:**
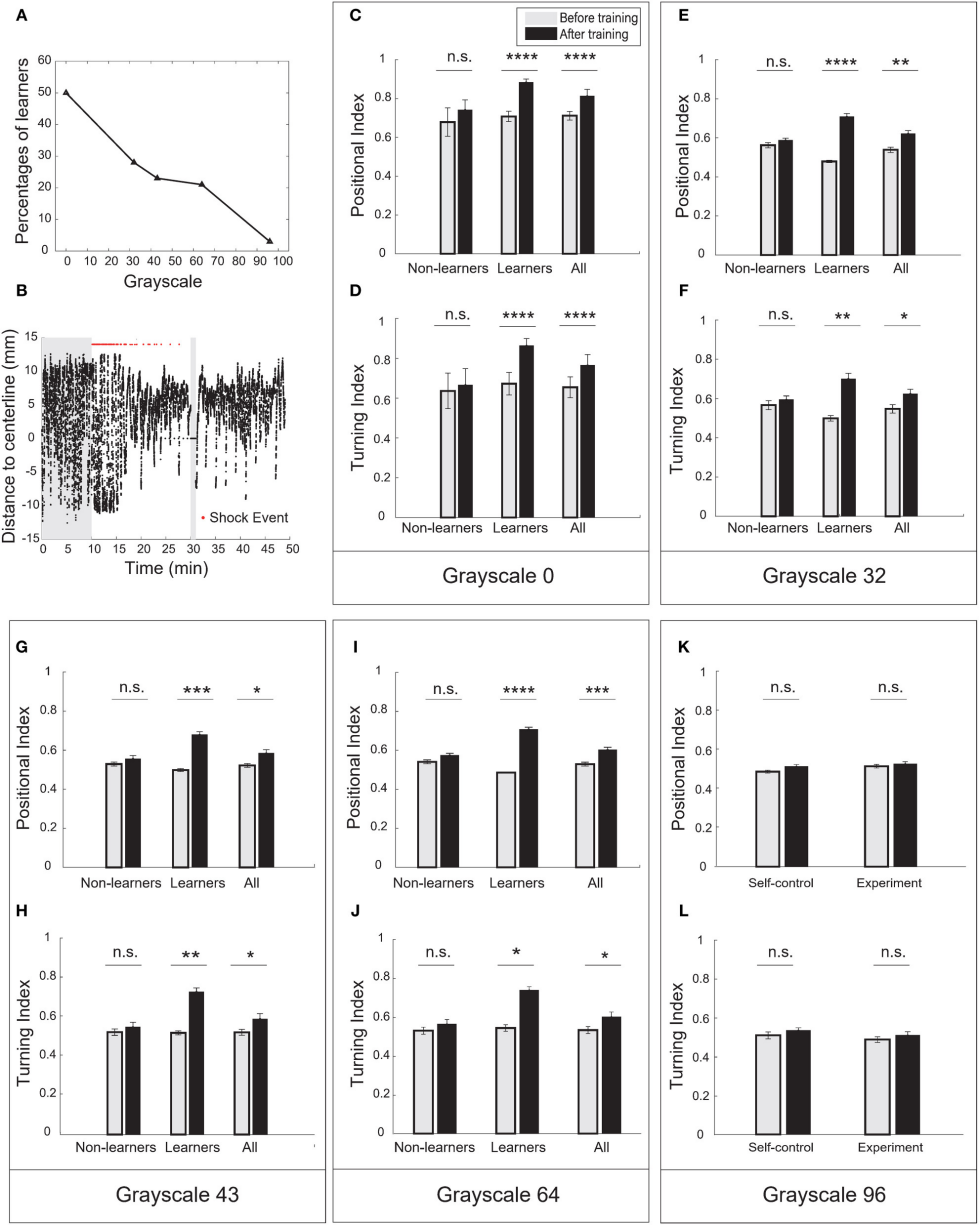
Visual contrast was the key parameter to modulate operant learning responses. **(A)** The percentage of learners decreased with the grayscale value of conditioned patterns. **(B)** A typical learner's behavioral trace. Pure-black (grayscale-0) was used as the conditioned pattern. **(C)** Analysis of the positional index (*t*-test, *p* = 1.98e-11 for the learners, *p* = 0.9492 for the non-learners, and *p* = 2.03e-06 for all fish). Pure-black (grayscale-0) was used as the conditioned pattern. **(D)** Learners also showed significant increase in the turning index (*t*-test, *p* = 1.22e-5 for learners, *p* = 0.6491 for non-learners, and *p* = 0.0057 for all fish). **(E)** Analysis of the positional index (*t*-test, *p* < 0.00001 for learners, *p* = 0.3252 for non-learners, and *p* = 0.0018 for all fish). Grayscale-32 was the conditioned pattern. **(F)** Learners also showed significant increase in the turning index (*t*-test, *p* = 0.0049 for learners, *p* = 0.4923 for non-learners, and *p* = 0.0331 for all fish). **(G)** Analysis of the positional index (*t*-test, *p* = 0.0005 for learners, *p* = 0.3182 for non-learners, and *p* = 0.0128 for all fish). Grayscale-43 was the conditioned pattern. **(H)** Learners also showed significant increase in the turning index (*t*-test, *p* = 0.0022 for learners, *p* = 0.5067 for non-learners, and *p* = 0.0498 for all fish). **(I)** Analysis of the positional index (*t*-test, *p* < 0.0001 for learners, *p* = 0.0927 for non-learners, and *p* = 0.0009 for all fish). Grayscale-64 was the conditioned pattern. **(J)** Learners also showed significant increase in the turning index (*t*-test, *p* = 0.0108 for learners, *p* = 0.3694 for non-learners, and *p* = 0.0480 for all fish). **(K)** Analysis of the positional index (*t*-test, *p* = 0.3130 for the experiment group, *p* = 0.0749 for the self-control group). Grayscale-96 was the conditioned pattern. **(L)** Analysis of the turning index suggested that fish did not show significant learning responses (*t*-test, *p* = 0.3360 for the experiment group, *p* = 0.3019 for the self-control group). All error bars are SEM. *p < 0.05, **p < 0.01, ***p < 0.001, ****p < 0.0001.

In the case of pure-black (grayscale-0), half of the fish population (21 out of 42, Table 2) can be classified as learners. They all showed significant increase in the positional index and the turning index after training (Figures 3C,D, Supplementary Figures 1A,B). Figure 3B showed a typical behavioral trace of a learner (see also Supplementary Videos 3, 4).

In the case of grayscale-96, however, fish showed little increase in the positional and turning indices (Figures 3K,L). We found only one fish can be classified as learner. In the other cases, a fraction of fish population exhibited learning responses (Figures 3E–J, Supplementary Figures 1C–H). The percentage of learners decreased with grayscale value (Figure 3A). Reversing the conditioned pattern and the background pattern in our paradigm, however, failed to elicit learning responses.

The red-black checkerboard and the grayscale-43 conditioned pattern had the same mean grayscale value (see Table 1). Consistently, similar percentages of fish (26 vs. 23%, Table 2) could learn the two conditioned patterns, respectively. Together, these results suggest that the visual contrast, not spatial checkerboard features, contributed to the operant learning responses.

### Visual Contrast Modulates Memory Extinction in the Operant Conditioning Task

To investigate how the operant conditioning behavior changed over time, we divided the entire process into epochs (excluding the blackout phase). Every 2-min interval is one epoch. The baseline phase has five epochs; the training phase has 10 epochs; and the test phase has nine epochs.

We defined the memory extinction point as the first time when the positional index within an epoch dropped below the mean index in the baseline phase; we defined the duration from the start of the test phase to the extinction point as the memory length. Memory length shorter than two epochs (e.g., fish may stay still in the non-CS zone) were excluded (see Materials and Methods).

**Figure 4:**
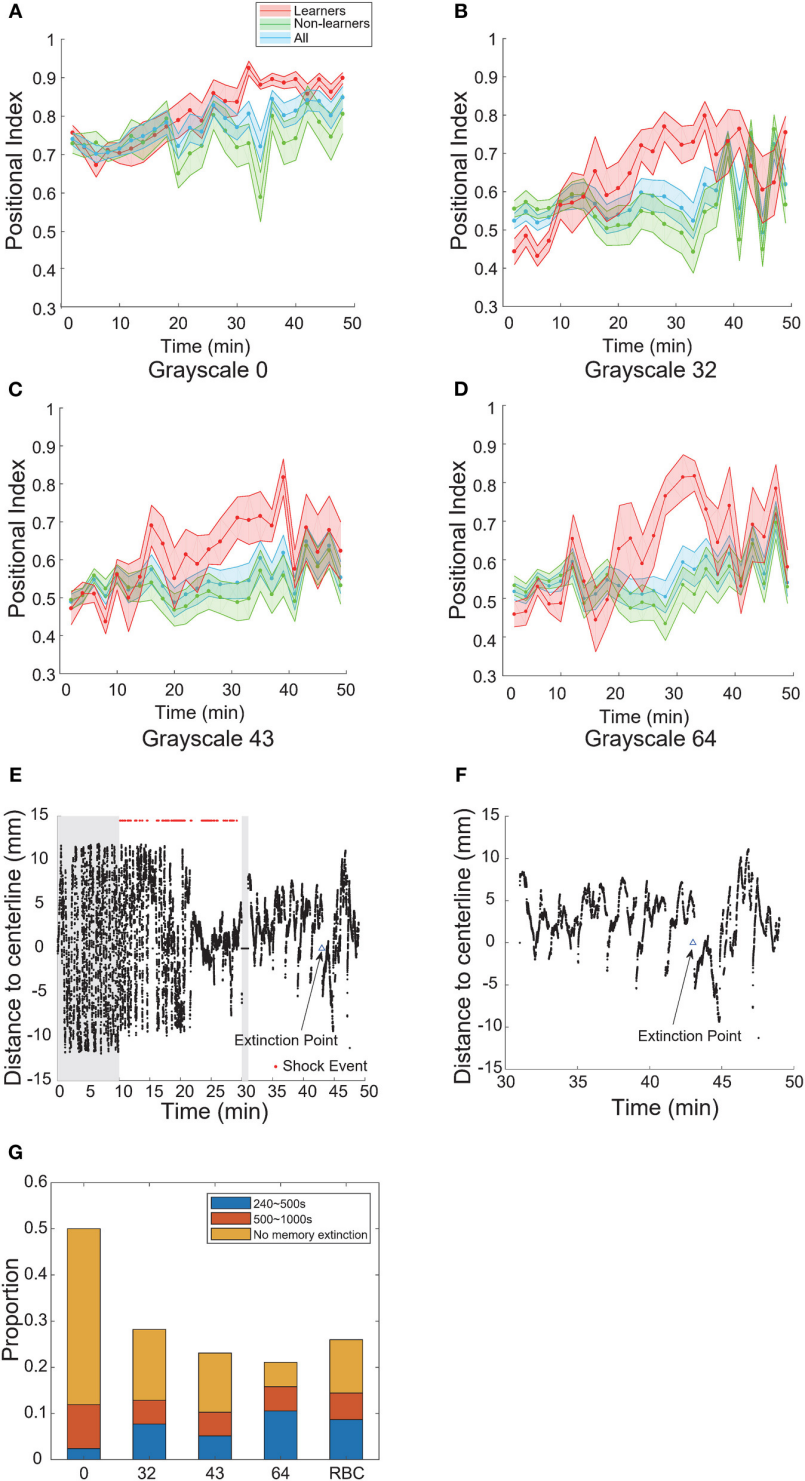
Visual contrast modulated memory extinction. **(A–D)** Learning curves for different grayscale conditioned patterns. **(E)** A typical learner's behavioral trace. Grayscale-32 was the conditioned pattern. The blue triangle indicates the extinction point. **(F)** A magnification of the test phase in **(E)**. **(G)** Memory length distribution for each conditioned pattern (grayscale values 0, 32, 43, 64, 96; RBC: red-black checkerboard). Blue: short-term memory fish (240–500 s); orange: mid-term memory fish (500–1,000 s); yellow: fish that did not show memory extinction during the entire test phase. All error bars are SEM.

We plotted the learning curves—the positional index vs. time—for learners and non-learners (Figures 4A–D). In the case of pure-black, the learning curve of learners rose and approached the maximum near the end of training; during the test phase, the learning curve remained high across the entire test phase (Figure 4A). Note that a large percentage of fish (16/21) did not show memory extinction (Figure 4G).

In the cases of other grayscale conditioned patterns, the learning curves of learners also reached their peaks near the end of training. However, all curves decayed after several epochs in the test phase (Figures 4B–D). The memory extinction points were similar (grayscale-32 at 45 min, grayscale-43 at 41 min, grayscale- 64 at 41 min). Figures 4E,F illustrate a typical animal that learned the association and then experienced memory extinction in the test phase. The fish started swimming more in the CS-zone near 43 min.

In Figure 4G, we compared the distribution of memory lengths across all grayscale conditioned patterns. The mean memory length was the highest (970 s, Table 4) and the rate of extinction (see Materials and Methods) was the lowest in the case of pure-black conditioned pattern (Table 3).

**Table 3:** Memory extinction rates.

	**Grayscale 0**	**Grayscale 32**	**Grayscale 43**	**Grayscale 64**	**Red-black checkerboard**
*elavl3 (min^−1^)*	3.83e-3	4.68e-3	1.00e-3	3.00e-2	8.11e-3
*AB/WT (min^−1^)*	9.82e-3	8.33e-3	1.01e-2	2.01e-2	2.04e-2

**Table 4:** Mean memory lengths of learners.

	**Grayscale 0**	**Grayscale 32**	**Grayscale 43**	**Grayscale 64**	**Red-black checkerboard**
*elavl3 (s)*	970	807	813	570	756
*AB/WT (s)*	725	690	744	665	691

We also found that the mean memory lengths (756 vs. 813 s) were similar when the red-black checkerboard and grayscale-43 pattern were used as the CS (Table 4). The percentages of learners that did not show memory extinction were also similar (12/27 vs. 5/9) in the two groups.

### Wild-Type Fish Show Similar Learning Responses in the Operant Conditioning Task

**Figure 5:**
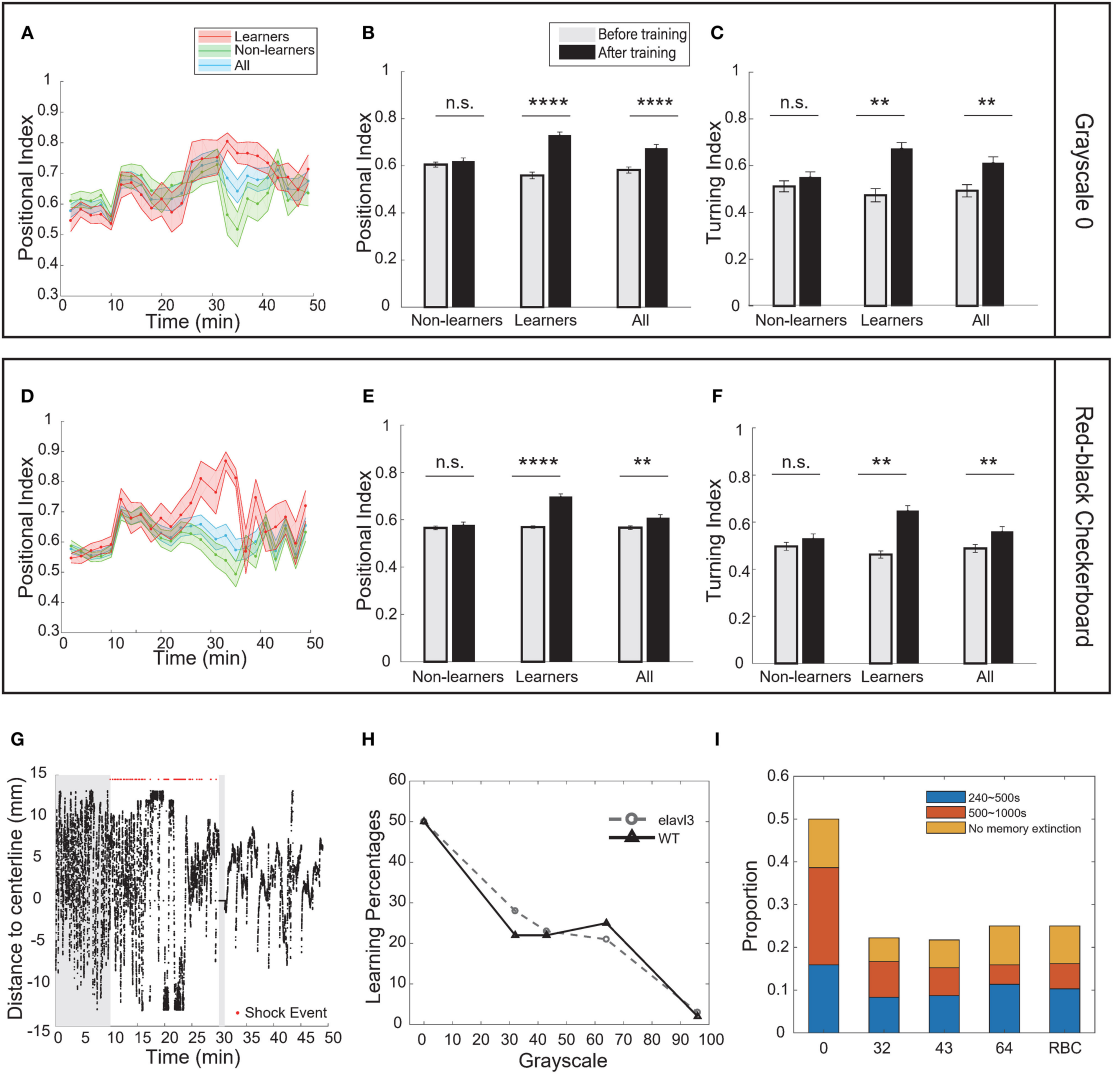
Strain differences in the operant learning task. **(A)** Learning curves of wild-type (AB) fish. Pure-black was the conditioned pattern. **(B)** Analysis of the positional index of wild-type fish presented with the pure-black conditioned pattern (*t*-test, *p* < 0.0001 for learners, *p* = 0.5672 for non-learners, and *p* < 0.0001 for all fish). **(C)** Learners also showed significant increase in the turning index (*t*-test, *p* = 0.0082 for learners, *p* = 0.4561 for non-learners, and *p* = 0.0094 for all fish). **(D)** Learning curves of wild-type fish. Red-black checkerboard was the conditioned pattern. **(E)** Analysis of the positional index of wild-type fish presented with the red-black checkerboard conditioned pattern (*t*-test, learners: *p* = 0.0001; non-learners: *p* = 0.4768; all: *p* = 0.0082). **(F)** Wild-type learners also showed significant increase in the turning index (*t*-test, *p* = 0.0035 for learners, *p* = 0.2514 for non-learners, and *p* = 0.0087 for all fish). **(G)** A typical wild-type learner's behavioral trace. **(H)** Percentage of learners vs. the grayscale value of conditioned patterns for transgenic (elavl3) and wild-type fish. **(I)** Memory length distribution for wild-type fish. All error bars are SEM. **p < 0.01, ****p < 0.0001.

To test whether the learning effect was strain-specific, we also performed the operant conditioning task in AB wild-type fish. Like transgenic fish, the percentage of learners in AB fish also decreased with the grayscale value of conditioned patterns (Figure 5H, Supplementary Figures 2C–H, 3): half of the fish population could be classified as learners in the case of pure-black (Figures 5A–C, Supplementary Figures 2A,B); whereas only one in the case of grayscale-96 (Supplementary Figures 2I,J). Some AB fish can learn the association between the red-black checkerboard and US (Figures 5D–F, Supplementary Figures 2K,L), but not between the white-black checkerboard and US (Supplementary Figures 2M,N).

We note that in the case of pure-black CS, more wild-type fish exhibited memory extinction (Figure 5I). Figure 5G shows a learner with memory extinction in the test phase. Fish started to swim more in the CS zone at 41 min.

## Discussion

### Operant Learning in Larval Zebrafish

Operant learning allows animals to avoid danger or to find potential reward in a complex environment (Skinner, 1984). An earlier study (Li, 2012) demonstrated an operant learning paradigm for head-fixed zebrafish larvae, where ~75% larvae learned to correlate a positive consequence—the relief of heat exposure—with biased tail turning in the absence of conditioned cue.

In the current work, we demonstrated that a significant proportion of 7–10 dpf larval zebrafish showed significant operant learning responses when a darker-than-background conditioned pattern, such as a pure-black pattern, was paired with a punishment—moderate electroshock. In an earlier study (Valente et al., 2012), it was reported that 1-week larvae showed no significant learning response. Several factors may explain the discrepancy.

First, we observed little learning response when the white-black checkerboard or grayscale-96 pattern was paired with the US (only one fish learned the contingency), consistent with Valente's result. Enhancement of learning was observed, however, when a darker visual pattern, such as a pure-black pattern, was paired with the US.

Second, in our modified paradigm, fish possessed more opportunities to learn the contingency between the CS and US during the training period: when fish stayed in the non-CS zone for more than 48 s, the positions of CS and non-CS patterns would update. In Valente's paradigm, however, no visual pattern updates were implemented when fish stayed within the non-CS zone.

### Visual Contrast Is the Key Parameter to Modulate Operant Learning Responses

Larval zebrafish can detect light intensity change at a very early age (Easter and Nicola, 1996; Emran et al., 2008). Opsins that are sensitive to long and short wavelength light start to be expressed in cone photoreceptors at 50 hpf (Raymond et al., 1995). During optomotor behaviors, 7 dpf larvae can detect motion stimuli by computing the spatiotemporal correlation of light intensities from nearby pixels in a visual pattern (Orger et al., 2000; Orger and Baier, 2005). Here, our study shows that the visual contrast, rather than spatial features in a visual pattern, is critical in modulating larval zebrafish operant learning responses.

Many studies have demonstrated that larval zebrafish exhibit positive phototaxis (Steenbergen et al., 2011; Chen and Engert, 2014; Guggiana-Nilo and Engert, 2016). In our behavioral paradigm, the behavioral metric baselines (e.g., positional index) were computed first before the training phase (see Materials and Methods). Light intensity could shift the baselines due to an innate illuminance bias. Significant changes in the behavioral metrics during and after operant conditioning (see Figure 2), however, require an explanation that goes beyond the innate avoidance response.

Here we speculate that this visual-contrast-dependent learning may arise from the crosstalk between phototaxis and fear conditioning circuits. Both phototaxis and US-triggered fear responses involve habenula (Agetsuma et al., 2010; Zhang et al., 2017), a specialized brain region where a direct association between a CS and fear may occur through synaptic plasticity. According to this model, a CS would trigger fear responses, and learning leads to a stronger association between visual-related inputs and escape responses. These predictions can potentially be tested by combining our behavioral system with whole brain calcium imaging in freely behaving larval zebrafish (Cong et al., 2017).

### Memory Extinction in the Operant Conditioning Task

Memory extinction is an active learning process where an animal learns to dissociate the conditioned response and CS in the absence of US (Myers and Davis, 2007). In our assay, the extinction point was defined as the first epoch whose positional index dropped below the mean index of the baseline. In addition, fish that did not keep a high level of positional index for at least two epochs were not counted as learners (see Materials and Methods). When the pure-black pattern was used, few learners showed memory extinction before the test phase ended. The distribution of memory length (Figure 4G) is consistent with that in a recent classical conditioning paradigm in larval zebrafish (Aizenberg and Schuman, 2011). When other grayscale patterns were used as the CS, mean memory lengths were further reduced (Table 4).

We also computed the rate of memory extinction (see Materials and Methods) for different CS patterns. We found that the extinction rate increased with the grayscale value of conditioned patterns for both wild-type and transgenic strains (Table 3). Also, the extinction rate in wild-type strain was higher than that in the transgenic strain when grayscale-0 was the CS (Figures 4G, 5I).

The above observations suggest that the dissociation between conditioned patterns and behavior could also be modulated by the visual contrast. Recent studies in Drosophila (Felsenberg et al., 2017, 2018) showed that aversive memory formation and extinction involved different neural circuits and they competed with each other during decision-making. Identifying the neuronal circuitries underlying memory representation and extinction might help us test the hypothesis.

### Strain Differences in the Operant Conditioning Task

We performed operational conditioning for both *elavl3:H2B-GCaMP6f* (in the *casper* mutant background) transgenic fish and wild-type (AB) fish and found that visual contrast modulated learning behaviors in similar ways (Figure 5). Nevertheless, more wild-type fish exhibited memory extinction when the pure-black conditioned pattern was used. A previous study (Parker et al., 2013) has systematically characterized the behaviors of wild-type (TU) fish and *casper* mutants. No difference was found in adult fish, yet wild-type fish showed a significant increase in anxiety when treated with 1-pheyl-2-thiourea (PTU), a drug that is used to maintain the transparency of embryos. Whether differences in the internal state of the two strains led to the difference of memory extinction in our behavioral paradigm requires further investigation.

### High-Throughput Behavioral Assays for Learning and Memory in Larval Zebrafish

Larval zebrafish are amenable to high-throughput screen due to their transparency, small size and high permeability to small molecules (Kokel et al., 2010; Rihel et al., 2010). Though most systems are designed for drug or genetic screens (Rihel et al., 2010; Gehrig et al., 2018; Yang X. et al., 2018), here we have developed a high-throughput behavioral training system with custom supported software suites. Compared with previous work (Pelkowski et al., 2011; Hinz et al., 2013), the BLITZ software has enabled a fully automatic control of video capture, online image processing, visual pattern presentation and electroshocks delivery, making it an easily adaptable system for various purposes. Our complementary ABLITZER software also allows users to import, analyze and visualize data with well-structured classes and functions.

In its current version, our system cannot deal with situations of overlapping larvae, whose identities are hard to assign based on the current tracking algorithm. An earlier work (Mirat et al., 2013) showed that accurately tracking multiple larvae in groups over long periods of time were feasible. Integration of their algorithm with BLITZ may allow the study of social interactions of larval zebrafish in the future (Buske and Gerlai, 2014).

In conclusion, we have developed a high-throughput operant conditioning system for larval zebrafish. When using electroshocks as the US and the red-black checkerboard or pure-black pattern as the CS, we found that a significant portion of larval zebrafish population could acquire operant learning. We also compared the learning responses between AB wild-type fish and transgenic fish. The percentage of learners and memory length strongly depended upon the visual contrast. Future work that combines imaging and genetic tools should help identify neural mechanisms underlying the visual-contrast-dependent learning behavior.

## Author Contributions

WY conceived the study, designed, and built the behavioral setup, developed the software suites, designed, carried out the experiments, wrote the manuscript, and conceived the figures. YM helped carry out the experiment and conceive the figures. DL helped design, build the behavioral setup, and conceive the figures. QW helped design the experiments and wrote the manuscript.

### Conflict of Interest Statement

The authors declare that the research was conducted in the absence of any commercial or financial relationships that could be construed as a potential conflict of interest.

## Abbreviations

dpf: days post-fertilization
fps: frames per second
SEM: standard error of the mean
BLITZ: behavioral learning in the zebrafish
ABLITZER: the analyzer of BLITZ results.
