# Supplementary material for: Visual Contrast Modulates Operant Learning Responses in Larval Zebrafish
Source: Front Behav Neurosci. 2019 Jan 24;13:4. doi: 10.3389/fnbeh.2019.00004 (PMC6353835; doi:10.3389/fnbeh.2019.00004)
Supplement: Supplementary file 5 [file Data_Sheet_1.docx]

Supplementary Material

Visual intensity ratio modulates operant learning responses in larval zebrafish

Wenbin Yang^1,2*^, Yutong Meng^1,2^, Danyang Li^1,2^, Quan Wen^1,2,3*^

*** Correspondence:**
Wenbin Yang, Quan Wen
young24@mail.ustc.edu.cn, [qwen@ustc.edu.cn](mailto:qwen@ustc.edu.cn)


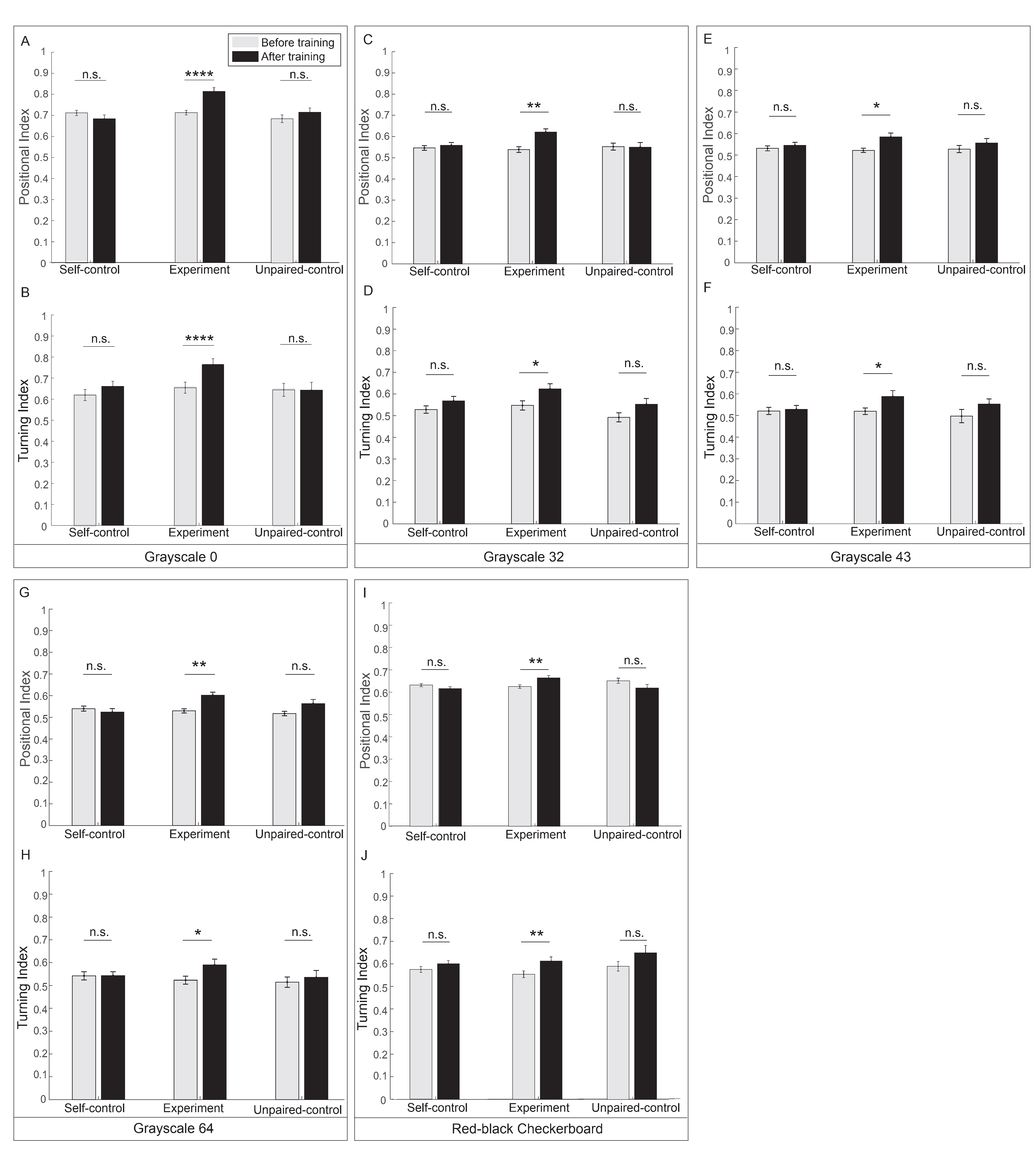


**Supplementary Figure 1. Self-control and unpaired-control results for conditioned patterns presented to *elavl3* transgenic fish**

1. In the case of grayscale-0 conditioned pattern, the positional index analysis suggests that fish showed no learning responses in the two control settings (t-test p = 0.2018 (N = 42); p < 0.0001 (N = 42); p = 0.1923 (N = 31), from left to right respectively, t-test).
2. Neither does the turning index analysis suggest that fish showed learning responses in the two control settings (t-test, p = 0.2811, p= 0.0057, p = 0.9837).
3. Analysis of the positional index (grayscale-32 conditioned pattern, t-test, p = 0.4874 (N = 39), p= 0.0018 (N = 39), p = 0.9321 (N = 19)).
4. Analysis of the turning index (grayscale-32, t-test, p = 0.1656, p= 0.0331, p = 0.0955).
5. Analysis of the positional index (grayscale-43, t-test, p = 0.4890 (N = 39), p= 0.0128 (N = 39), p = 0.3604 (N = 24)).
6. Analysis of the turning index (grayscale-43, t-test, p = 0.7483, p= 0.0498, p = 0.2166).
7. Analysis of the positional index (grayscale-64, t-test, p = 0.4661 (N = 38), p= 0.0009 (N = 38), p = 0.0808 (N = 19)).
8. Analysis of the turning index (grayscale-64, t-test, p = 0.9703, p= 0.0480, p = 0.6100).
9. Analysis of the positional index (RBC, t-test, p = 0.0843 (N = 104), p= 0.0021 (N = 104), p = 0.1260 (N = 28)).
10. Analysis of the turning index (RBC, t-test, p = 0.1836, p= 0.0099, p = 0.0628). All error bars are SEM.


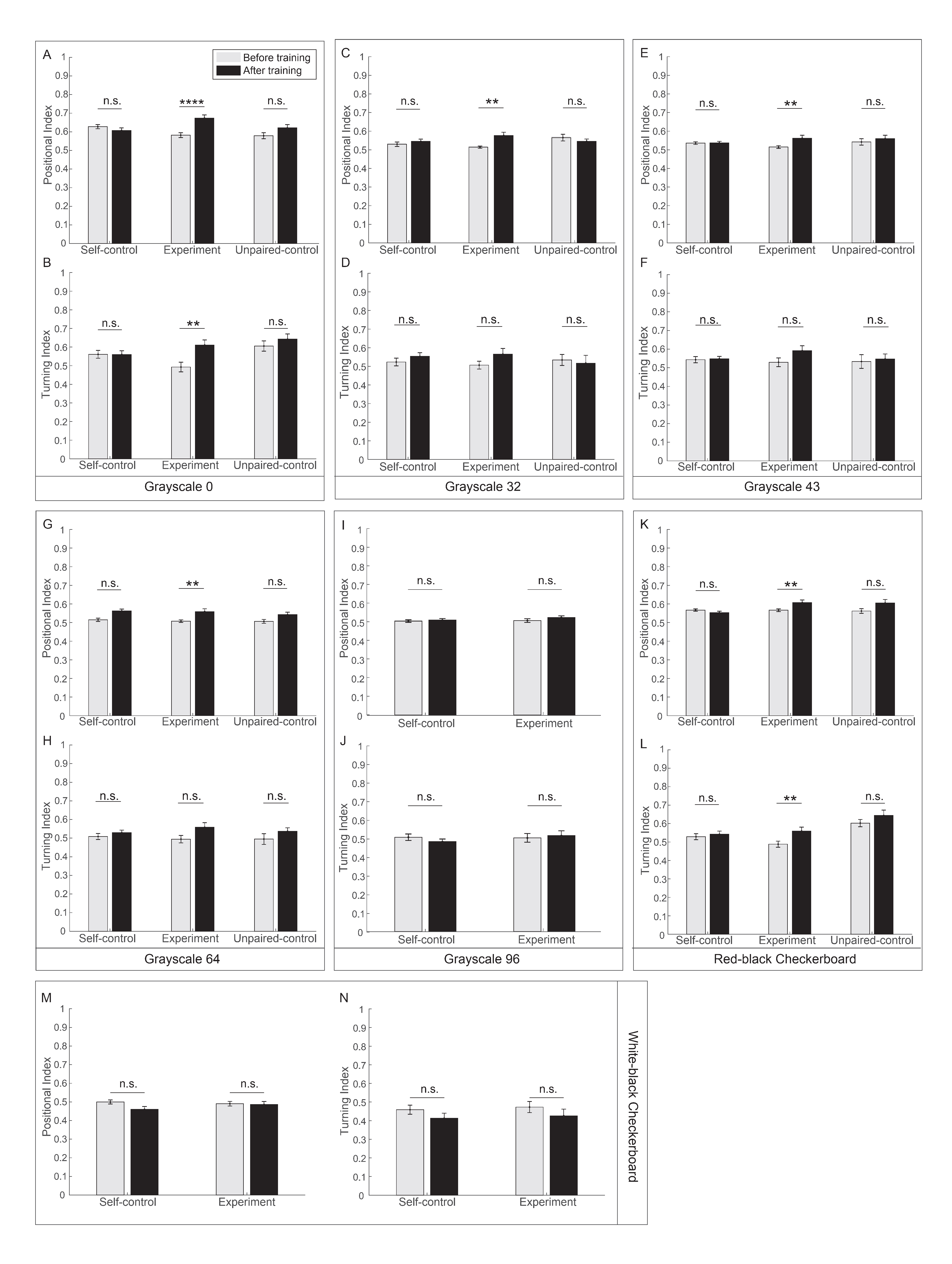


**Supplementary Figure 2. Self-control and unpaired-control results for conditioned patterns used in AB wild type fish**

1. In the case of grayscale-0 conditioned pattern, the positional index analysis suggests that fish showed no significant learning responses in the two control settings (t-test, p = 0.0796 (N = 44), p < 0.00001 (N = 44), p = 0.0625 (N = 24), from left to right respectively).
2. Neither does the turning index analysis suggests that fish showed significant learning responses in the two control settings (t-test, p = 0.9664, p= 0.0094, p = 0.3655).
3. Analysis of the positional index (grayscale-32 conditioned pattern, t-test, p = 0.3388 (N = 36), p= 0.0023 (N = 36), p = 0.5078 (N = 18)).
4. Analysis of the turning index (grayscale-32, t-test, p = 0.3116, p = 0.1142, p = 0.7676).
5. Analysis of the positional index (grayscale-43, t-test, p = 0.9177 (N=46), p = 0.0099 (N=46), p = 0.4232 (N =24)).
6. Analysis of the turning index (grayscale-43, t-test, p = 0.8198, p= 0.0748, p = 0.7324).
7. Analysis of the positional index (grayscale-64, t-test, p = 0.4580 (N = 44), p= 0.0060 (N=44), p = 0.0746 (N = 19)).
8. Analysis of the turning index (grayscale-64, t-test, p = 0.2785, p= 0.0966, p = 0.2830).
9. In the case of grayscale-96 conditioned pattern, the positional index analysis suggests that fish did not show significant learning responses (t-test, p = 0.6168 (N = 41), p= 0.2067 (N = 41); There was no unpaired-control group because no significant learning responses were found in the experiment group).
10. Neither does the turning index analysis suggests that fish showed significant learning responses (t-test, p = 0.2831, p= 0.6857, p = 0.5753).
11. Analysis of the positional index (RBC, t-test, p=0.1332 (N = 68), p = 0.0082 (N = 68), p = 0.0921 (N = 22)).
12. Analysis of the turning index (RBC, t-test, p = 0.5555, p= 0.0087, p = 0.2454).
13. In the case of white-black checkerboard (WBC) conditioned pattern, the positional index analysis suggests that fish did not show significant learning responses (t-test, p = 0.0743 (N = 16), p= 0.8078 (N = 16)).
14. Neither does the turning index analysis suggests that fish showed significant learning responses (WBC, t-test, p = 0.2018, p= 0.4433). All error bars are SEM.


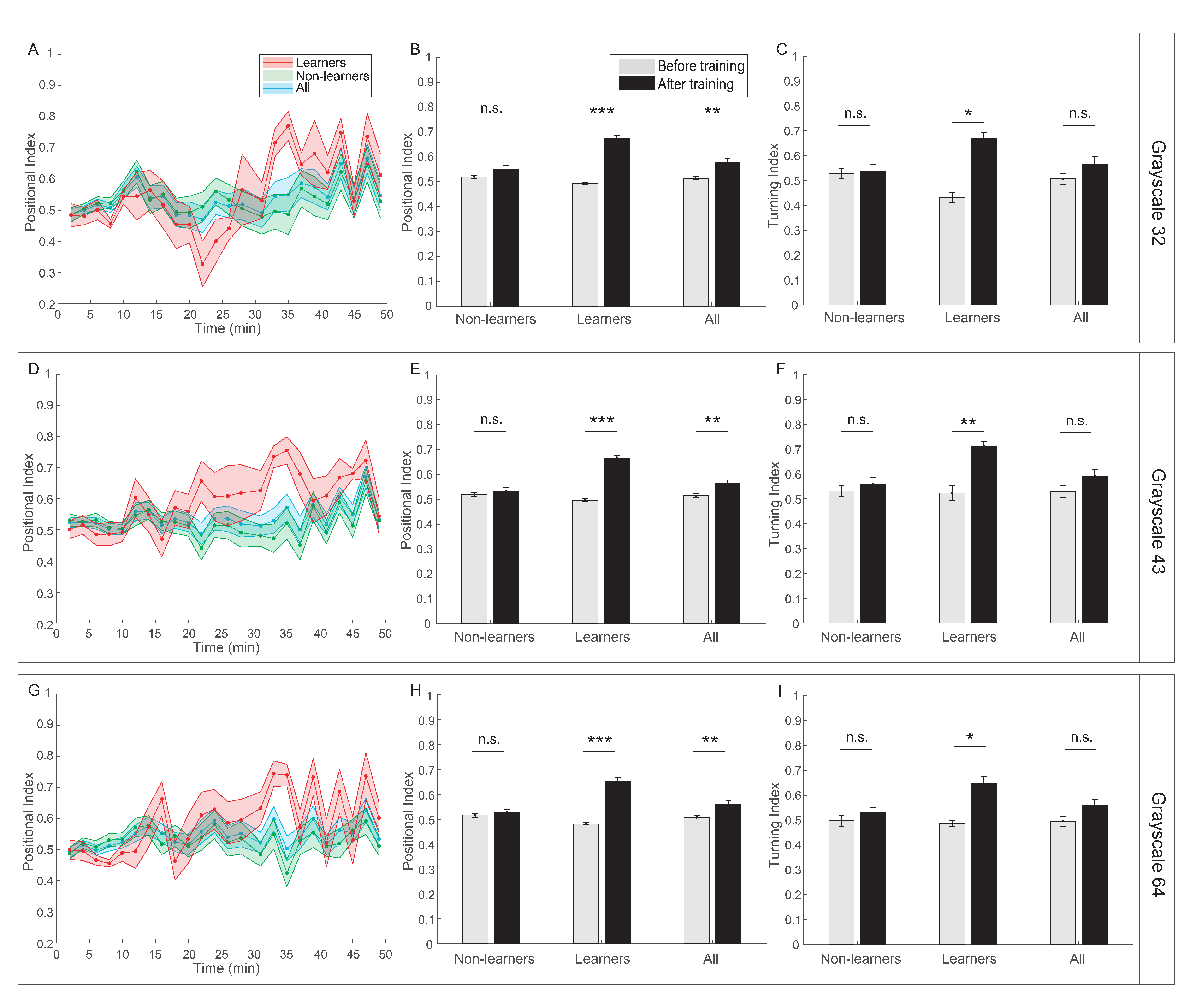


**Supplementary Figure 3. Operant learning responses in AB wild-type strain**

1. Learning curves of fish presented with the grayscale-32 conditioned pattern.
2. Analysis of the positional index (grayscale-32, t-test, p = 0.0004 for learners, p = 0.1340 for non-learners, and p = 0.0023 for all fish).
3. Learners also showed significant increment in the turning index (grayscale-32, t-test, p = 0.0124 for learners, p = 0.8279 for non-learners, and p = 0.1142 for all fish).
4. Learning curves of fish presented with the grayscale-43 conditioned pattern.
5. Analysis of the positional index (grayscale-43, t-test, p = 0.0001 for learners, p = 0.4430 for non-learners, and p = 0.0099 for all fish).
6. Learners also showed significant increment in the turning index (grayscale-43, t-test, p = 0.0087 for learners, p = 0.4957 for non-learners, p = 0.0748 for all fish).
7. Learning curves of fish presented with the grayscale-64 conditioned pattern.
8. Analysis of the positional index (grayscale-64, t-test, p = 0.0003 for learners, p = 0.4737 for non-learners, and p = 0.0060 for all fish).
9. Learners also showed significant increment in the turning index (grayscale-64, t-test, p = 0.0498 for learners, p = 0.4657 for non-learners p = 0.0966 for all fish). All error bars are SEM.


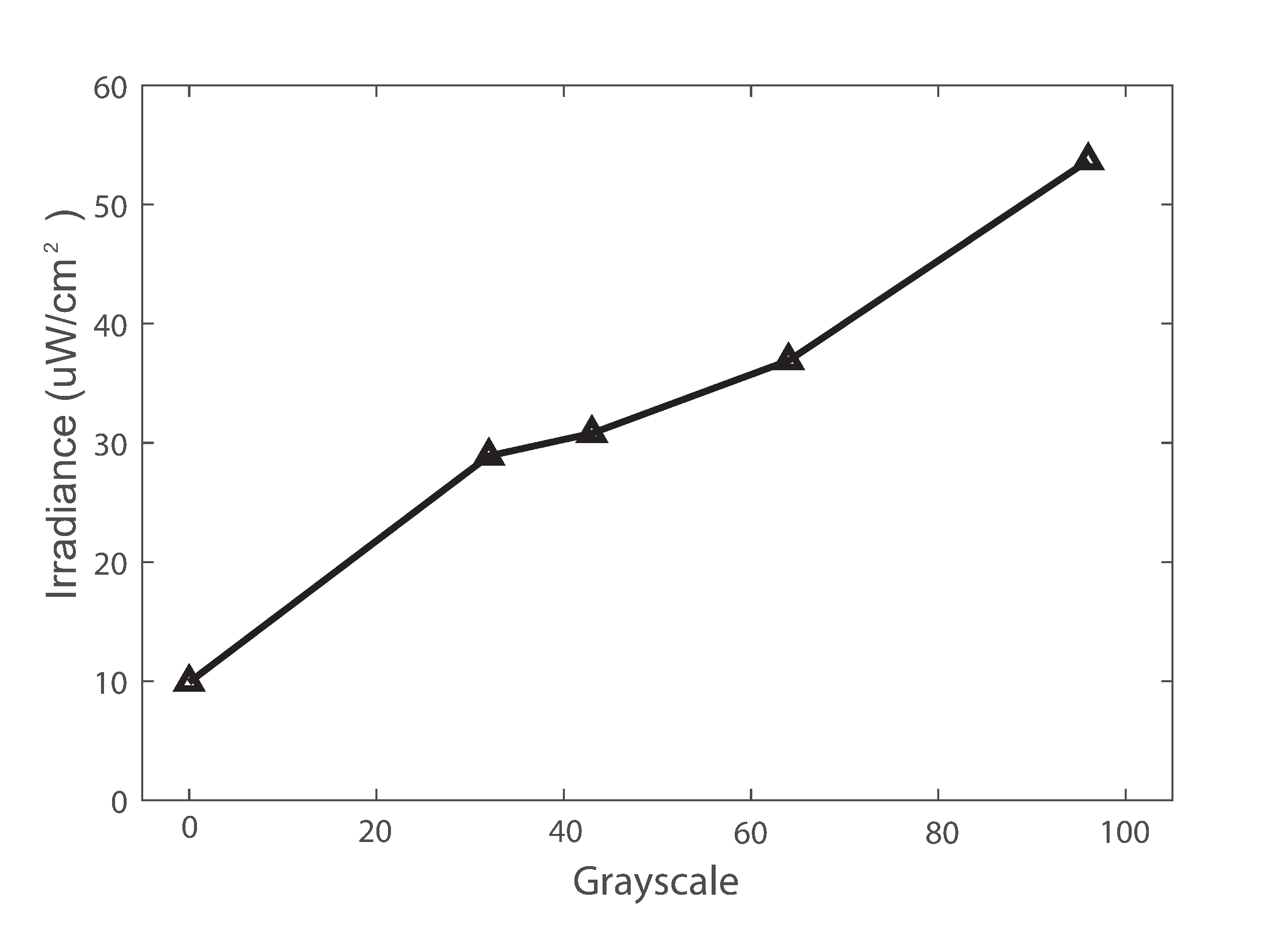


**Supplementary Figure 4. Measured light irradiance of conditioned patterns versus their grayscale values**


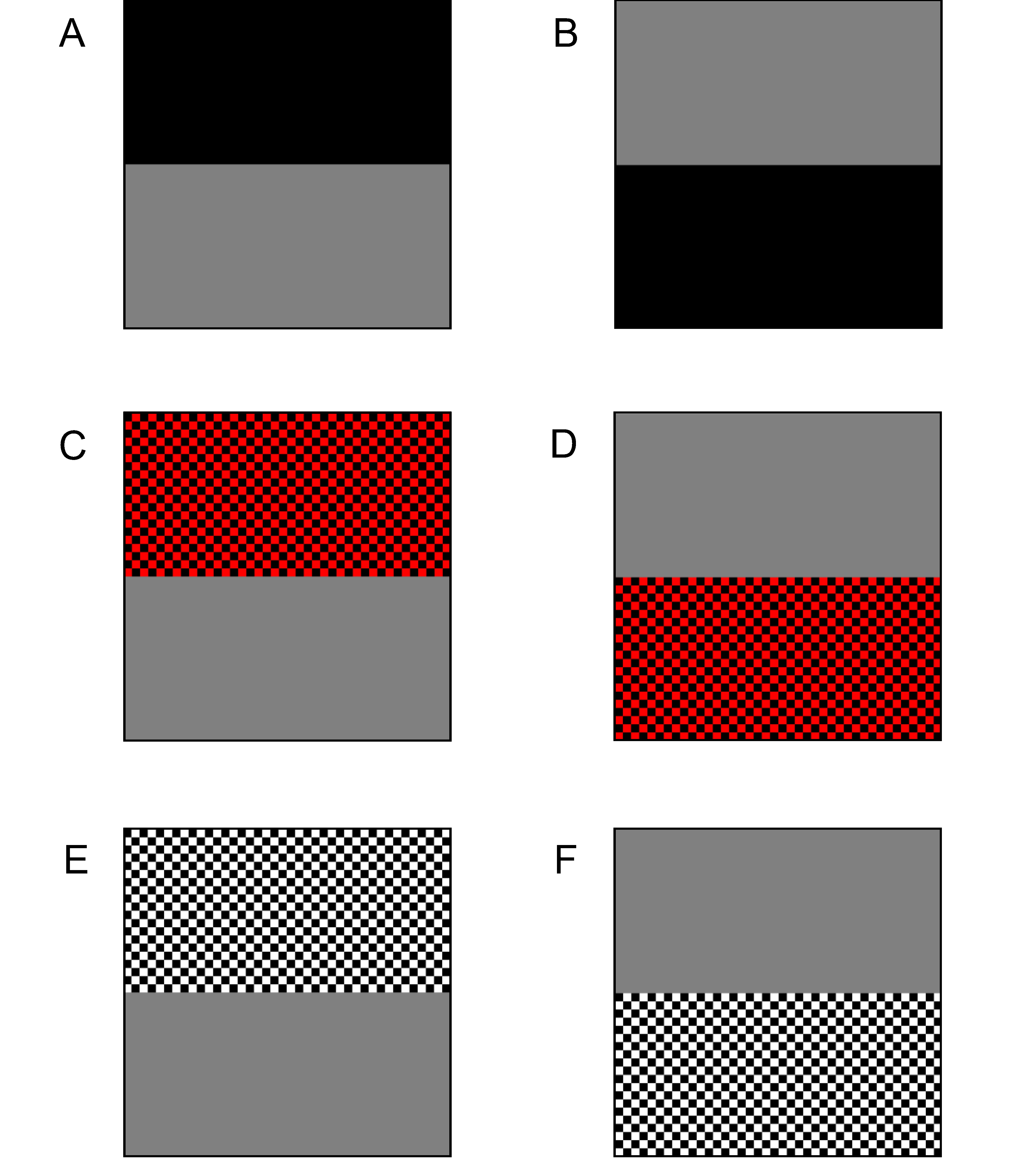


**Supplementary Figure 5. Representative visual patterns used in the operant conditioning task**

(A) In operant conditioning, the visual pattern with the pure-black pattern as the CS and the pure-gray pattern as the non-CS. (CS zone on the top)

(B) In operant conditioning, the visual pattern with the pure-black pattern as the CS and the pure-gray pattern as the non-CS. (CS zone on the bottom)

(C) In operant conditioning, the visual pattern with the red-black checkerboard as the CS and the pure-gray pattern as the non-CS. (CS zone on the top)

(D) In operant conditioning, the visual pattern with the red-black checkerboard as the CS and the pure-gray pattern as the non-CS. (CS zone on the bottom)

(E) In operant conditioning, the visual pattern with the white-black checkerboard as the CS and the pure-gray pattern as the non-CS. (CS zone on the top)

(F) In operant conditioning, the visual pattern with the white-black checkerboard as the CS and the pure-gray pattern as the non-CS. (CS zone on the bottom)


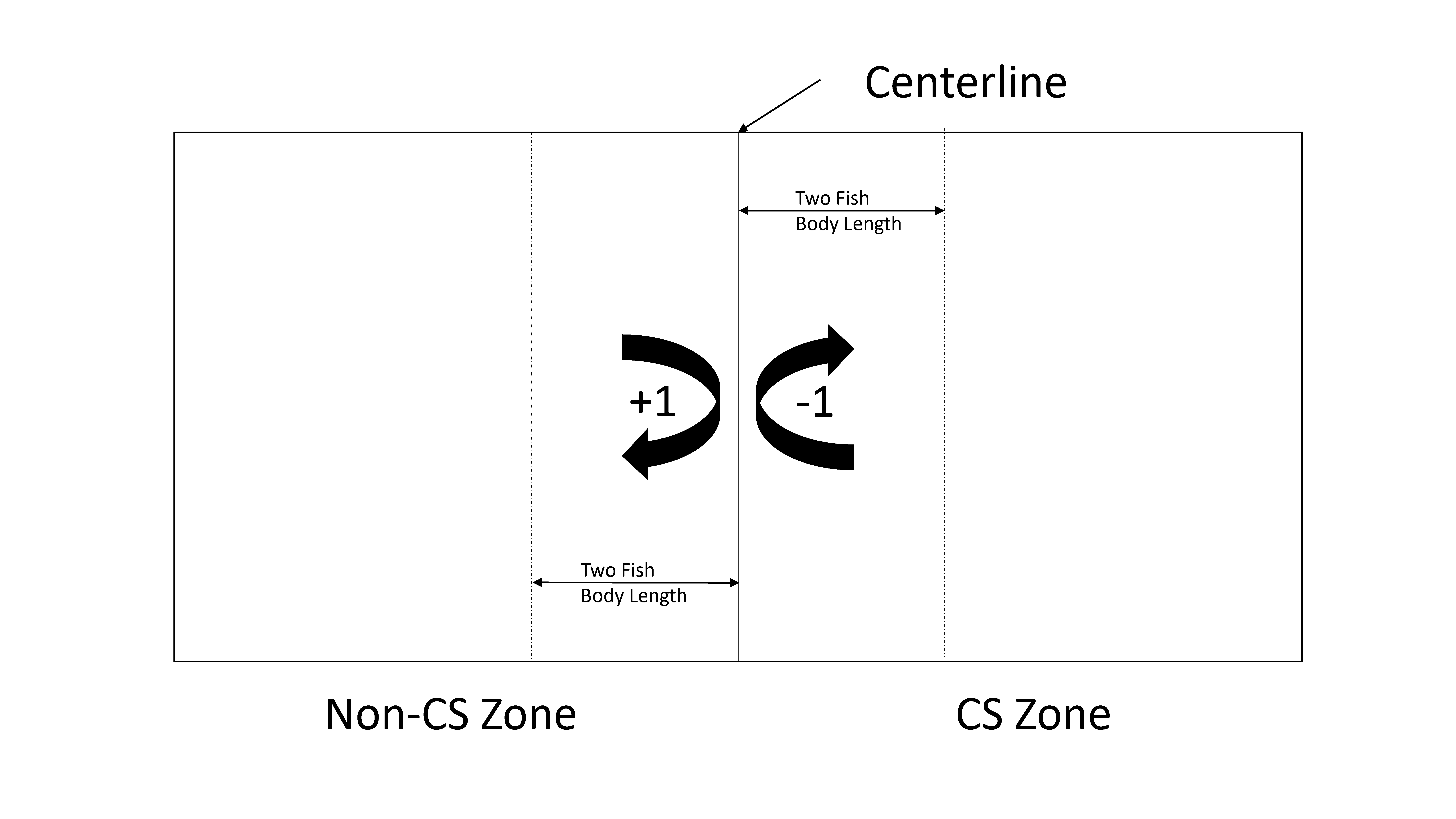


**Supplementary Figure 6. Calculation of the turning index**

Turning events were scored only when fish were within two-body-length from the midline. When fish turned away from the CS zone, +1 was scored; otherwise -1 was scored.


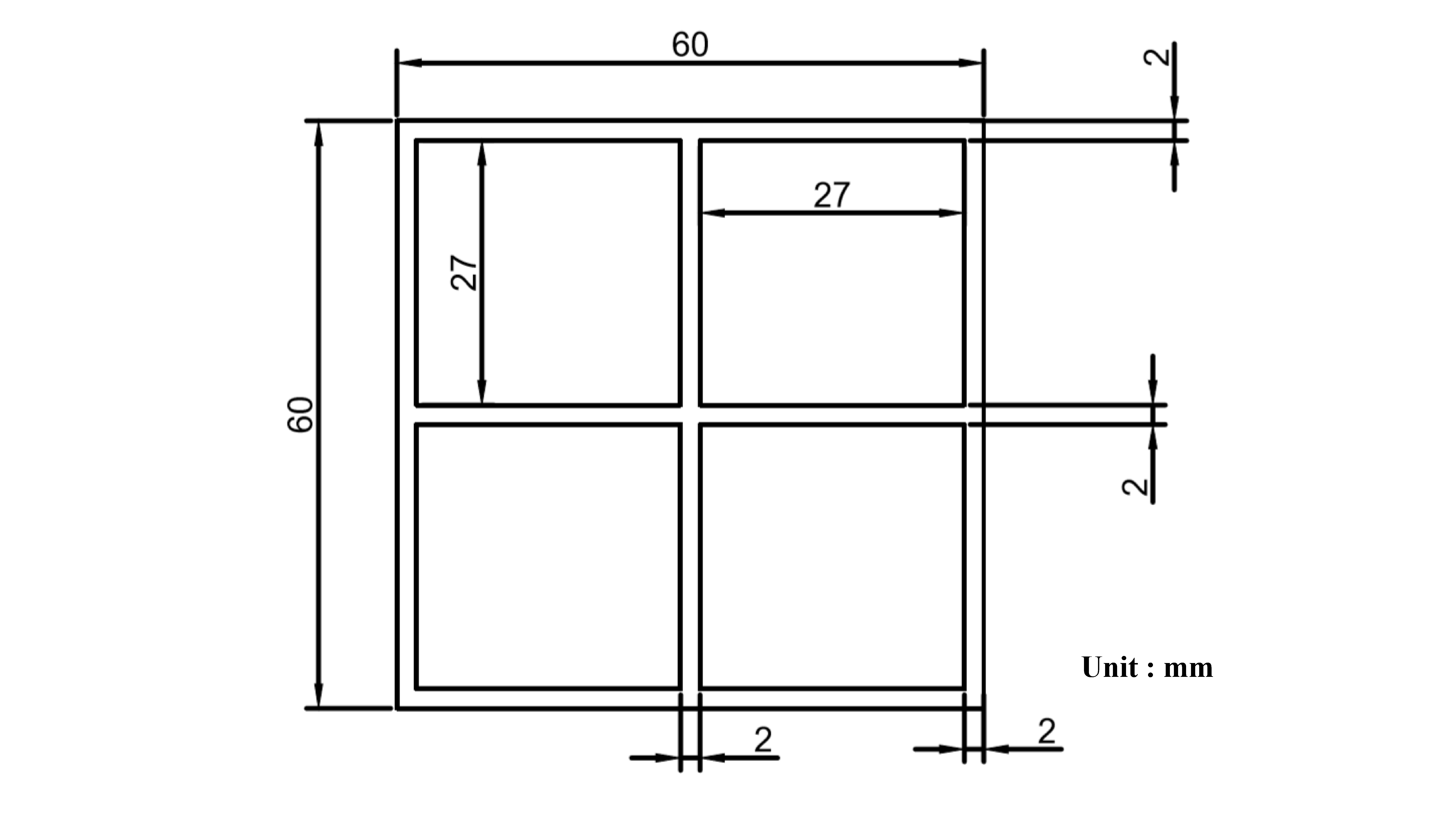


**Supplementary Figure 7. Fish container drawing**

The size of the arena is 27 x 27 (mm). Acrylic walls were blackened with permanent markers (Sharpie. Inc., US).

**Supplementary Video 1. Learner in the baseline phase of the operant conditioning with the red-black checkerboard as the CS.**

Locomotion of larvae (10 dpf) in the baseline phase of the operant conditioning. (Larvae’s head was denoted as a blue circle and the demarcating line is indicated as the red dashed-dot line.) The CS zone is the red-black checkerboard, the non-CS zone is the pure-gray pattern. The positions of zones interchanged for a random interval (uniformly sampled from 30 – 45 seconds) and no electroshock delivered. The CS zone position was annotated at the left-corner. The video plays at 3x speed as it was recorded.

**Supplementary Video 2. Learner in the test phase of the operant conditioning with the red-black checkerboard as the CS.**

Locomotion of larvae (10 dpf) in the test phase of the operant conditioning. (Larvae’s head was denoted as a blue circle and the demarcating line is indicated as the red dashed-dot line.) The CS zone is the red-black checkerboard, the non-CS zone is the pure-gray pattern. The positions of zones interchanged every two minutes and no electroshock delivered. The CS zone position was annotated at the left-corner. The video plays at 3x speed as it was recorded.

**Supplementary Video 3. Learner in the baseline phase of the operant conditioning with the pure-black pattern as the CS.**

Locomotion of larvae (7 dpf) in the baseline phase of the operant conditioning. (Larvae’s head was denoted as a blue circle and the demarcating line is indicated as the red dashed-dot line.) The CS zone is the pure-black pattern, the non-CS zone is the pure-gray pattern. The positions of zones interchanged for a random interval (uniformly sampled from 30 – 45 seconds) and no electroshock delivered. The CS zone position was annotated at the left-corner. The video plays at 3x speed as it was recorded.

**Supplementary Video 4. Learner in the test phase of the operant conditioning with the pure-black pattern as the CS.**

Locomotion of larvae (7dpf) in the test phase of the operant conditioning. (Larvae’s head was denoted as a blue circle and the demarcating line is indicated as the red dashed-dot line.) The CS zone is the pure-black pattern, the non-CS zone is the pure-gray pattern. The positions of zones interchanged every two minutes and no electroshock delivered. The CS zone position was annotated at the left-corner. The video plays at 3x speed as it was recorded.
